# Effect of Preoperative Home-Based Exercise Training on Quality of Life After Lung Cancer Surgery: A Multicenter Randomized Controlled Trial

**DOI:** 10.1245/s10434-023-14503-2

**Published:** 2023-11-07

**Authors:** Pedro Machado, Sara Pimenta, Ana Luís Garcia, Tiago Nogueira, Sónia Silva, Claúdia Lares dos Santos, Maria Vitória Martins, André Canha, Bárbara Oliveiros, Raul A. Martins, Joana Cruz

**Affiliations:** 1 Center for Innovative Care and Health Technology (ciTechCare), School of Health Sciences of the Polytechnic of Leiria (ESSLei), Leiria, Portugal; 2 University of Coimbra, Research Unit for Sport and Physical Activity (CIDAF, UID/PTD/04213/2019), Faculty of Sport Sciences and Physical Education, Coimbra, Portugal; 3 Physioclem, Physical Therapy Clinics, Alcobaça, Portugal; 4 Thoracic Surgery Unit, Portuguese Oncology Institute of Coimbra, Coimbra, Portugal; 5 Pulmonology Department, Leiria Hospital Center, Leiria, Portugal; 6 Pulmonology Department, District Hospital of Santarém, Santarém, Portugal; 7 Pulmonology Department, District Hospital of Figueira da Foz, Figueira da Foz, Portugal; 8 Physical Medicine and Rehabilitation Department, District Hospital of Santarém, Santarém, Portugal; 9 Laboratory of Biostatistics and Medical Informatics (LBIM), Faculty of Medicine, University of Coimbra, Coimbra, Portugal; 10 Faculty of Medicine, University of Coimbra, Coimbra Institute for Clinical and Biomedical Research (iCBR), Coimbra, Portugal; 11 Institute for Biomedical Imaging and Translational Research (CIBIT), University of Coimbra, Coimbra, Portugal

**Keywords:** Lung cancer, Surgical resection, Prehabilitation, Home-based exercise training, Quality of life

## Abstract

**Background:**

Preoperative exercise training is recommended for improvement of clinical outcomes after lung cancer (LC) surgery. However, its effectiveness in preventing postoperative decline in quality of life (QoL) remains unknown. This study investigated the effect of preoperative home-based exercise training (PHET) on QoL after LC surgery.

**Methods:**

Patients awaiting LC resection were randomized to PHET or a control group (CG). The PHET program combined aerobic and resistance exercise, with weekly telephone supervision. Primary outcome was QoL-assessed with the European Organization for Research and Treatment of Cancer (EORTC) Quality of Life Questionnaire C30 (QLQ-C30) at baseline, before surgery, and 1 month after surgery. The secondary outcomes were hospital length of stay and physical performance. The main analysis included a factorial repeated-measures analysis of variance. Additionally, the proportion of patients experiencing clinical deterioration from baseline to post-surgery was assessed.

**Results:**

The study included 41 patients (68.1 ± 9.3 years; 68.3% male) in the intention-to-treat analysis (20 PHET patients, 21 CG patients). A significant group × time interaction was observed for global QoL (*p* = 0.004). Between-group differences in global QoL were statistically and clinically significant before surgery (mean difference [MD], 13.5 points; 95% confidence interval [CI], 2.4–24.6; *p* = 0.019) and after surgery (MD, 12.4 points; 95% CI, 1.3–23.4; *p* = 0.029), favoring PHET. Clinical deterioration of global QoL was reported by 71.4% of the CG patients compared with 30 % of the PHET patients (*p* = 0.003). Between-group differences in favor of PHET were found in pain and appetite loss as well as in physical, emotional and role functions after surgery (*p* < 0.05). Compared with CG, PHET was superior in improving preoperative five-times sit-to-stand and postoperative exercise capacity (*p* < 0.05). No between-group differences in other secondary outcomes were observed.

**Conclusion:**

The study showed that PHET can effectively prevent the decline in QoL after LC surgery.

**Supplementary Information:**

The online version contains supplementary material available at 10.1245/s10434-023-14503-2.

Surgical resection is the standard therapy for early-stage lung cancer patients and an important treatment method for patients with stage IIIA disease, ensuring a 5-year overall survival of about 60–80 %.^1^ However, surgical resection has a detrimental impact on patients’ health-related quality of life (HRQoL), with a high prevalence of pain, fatigue, and dyspnea after surgery and most patients reporting a deterioration of their physical and role functions during the first postoperative month.^2–5^

In addition, although global quality of life (QoL) gradually returns to preoperative values by 3–6 months after surgery, a significant proportion of patients continue to experience functional limitations and symptoms of fatigue, pain, and dyspnea for 1–2 years after surgery.^3,4,6–8^ These short- and long-term deleterious effects of surgery highlight the need for supportive interventions aimed at improving or restoring postoperative HRQoL, which is essential for promoting patient-centered care.^2,4,8^

Exercise training is a promising non-pharmacologic intervention that has consistently shown positive effects on the HRQoL of cancer patients. It is the most effective treatment for cancer-related fatigue,^9^ and improves both physical and mental health.^10–12^

The current clinical guidelines for optimal perioperative care of lung cancer patients strongly recommend preoperative exercise training to improve postoperative outcomes.^13^ Moreover, a growing body of evidence shows that this intervention enhances exercise capacity and reduces the risk of postoperative pulmonary complications.^14–17^ Despite these benefits and the recommendations for routine evaluation of HRQoL in lung cancer care,^18^ the effects of preoperative exercise training on postoperative HRQoL remain unknown.^11,13,15, 17^

Furthermore, research on preoperative exercise training for lung cancer patients has been focused on facility-based exercise programs,^14,16,19–21^ although it is known that patients awaiting major cancer surgery prefer to exercise in their home setting,^22,23^ and that environmental barriers such as availability and transportation problems may hinder patients` access to prehabilitation.^23,24^

Therefore, the primary aim of this study was to evaluate whether preoperative home-based exercise training (PHET) can prevent decline in HRQoL after lung cancer surgery. The secondary purpose was to evaluate the effect of PHET on the postoperative hospital length of stay (LOS) and physical performance of patients undergoing lung cancer resection.

## Methods

### Trial Design

**Fig. 1:**
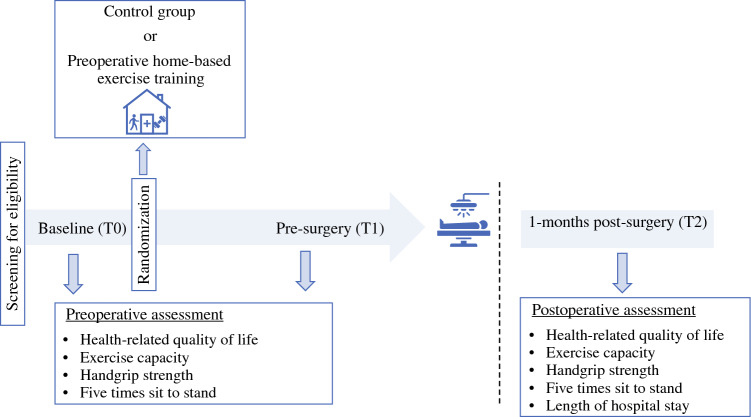
Study design

This multicenter, single-blind, parallel-arm, randomized controlled trial (RCT) (Fig. 1) recruited patients from the Portuguese Oncology Institute of Coimbra, Leiria Hospital Center, District Hospital of Santarém and District Hospital of Figueira da Foz (Portugal). The trial was registered at Clinicaltrials.gov (NCT05469425).

The study was conducted in accordance with the guidelines of the Declaration of Helsinki and approved by the Ethics Committee of the Leiria Hospital Center, District Hospital of Santarém, District Hospital of Figueira da Foz, and Portuguese Oncology Institute of Coimbra (protocol code TI 36/2021, 18 November 2021). This study is reported according to Consolidated Standards of Reporting Trials (CONSORT) guidelines.^25^

### Participants

Consecutive adult candidates for surgery (age ≥ 18 years) to treat confirmed or suspected lung malignancy (clinical stage IIIA or lower) who had medical approval for exercise and surgery scheduled for at least 2 weeks from the baseline assessment were considered for inclusion. The exclusion criteria ruled out metastatic tumor, contraindications for exercise training or physical testing (Table S1),^26–28^ inability to speak or understand Portuguese, and current involvement in regular exercise training (aerobic and resistance training during the past month ≥ 2 days per week, ≥ 30 min per session).

Potential participants were identified during routine appointments for pulmonology or thoracic surgery and invited to participate by their medical staff. If eligible, patients were contacted by the research team, who provided oral and written information about the trial. All patients who agreed to participate signed a written informed consent before any study assessment.

### Randomization and Blinding

Eligible patients were randomly assigned in a 1:1 ratio to either the PHET group or the control group (CG). Randomization was performed using a computerized random number generator^29^ with a random permuted block design (stratified by hospital site). Allocations were placed in consecutively numbered, opaque, and sealed envelopes,^29^ by an independent researcher who was not involved in patient recruitment or data collection. Group allocation was concealed until the baseline assessment was completed, after which the envelopes were opened in numeric order. Outcome assessors were blinded to group allocation and trained to perform the outcome assessments.

### PHET Group

The PHET program was previously tested in a feasibility trial,^30^ and is described following the Consensus on Exercise Reporting Template (Table S2).^31^ The length of the intervention was adjusted based on the waiting times for surgery and comprised three main components:

1. Educational session: During this session, a physical therapist instructed the patients about the importance of exercise training before lung cancer surgery, a factor that may increase exercise behavior,^23^ and demonstrated the correct technique of the home-based exercises. Additionally, patients were instructed on how to monitor training intensity using the Borg Category Ratio (Borg CR-10).^32^
2. Home-based aerobic plus resistance training (concurrent training): The rationale for prescribing concurrent training was based on guidelines for exercise prescription in oncology and on recent clinical trials that identified the combination of low-to-moderate-intensity aerobic plus resistance training as effective in improving HRQoL for cancer patients.^10,33^ The aerobic training consisted of walking thrice weekly, 30 min per session. Walking was chosen because it is the exercise modality preferred by most lung cancer patients.^34^ Its duration was increased to 40 min after the second week of the intervention. Resistance training was prescribed twice weekly on non-consecutive days and consisted of six exercises (Fig. S1), performed for two sets of 15 repetitions. The number of sets was increased to three sets per exercise after the second week of the intervention. The main goal of resistance training was to improve lower body functional strength because it has been associated with better HRQoL in cancer patients.^35^ Training intensity was prescribed based on the rating of perceived exertion (RPE) using the Borg CR-10 scale,^32^ a viable method for prescribing and monitoring training intensity in cancer patients.^10,36^ An RPE of 3–5 (moderate to strong) was recommended.
3. Telephone-based supervision: The PHET program was supervised through telephone calls by a physical therapist once a week.

### Control Group

The CG received the usual preoperative care offered at the health care units involved, which did not include structured exercise training. Additionally, the CG received weekly phone calls, which consisted of standardized questions regarding fatigue, pain, and dyspnea symptoms. Postoperatively, the participants in both the PHET and CG groups received standardized inpatient rehabilitation focused on early mobilization, breathing exercises, and incentive spirometry.

### Study Outcomes

The primary and secondary outcomes were assessed at three time points: T0 (baseline), T1 (before surgery, i.e., 1–5 days before surgery), and T2 (1 month after surgery).

### Primary Outcome: HRQoL

The study assessed HRQoL using the Portuguese version of the European Organization for Research and Treatment of Cancer (EORTC) Quality of Life Questionnaire C30 (QLQ-C30) version 3.0,^37,38^ as recommended by the international consensus on patient-centered outcomes for lung cancer.^18^ The Portuguese version of the questionnaire was previously validated with 933 cancer patients, revealing good psychometric properties.^37^

The QLQ-C30 includes a global QoL scale, five functioning scales, three multi-item symptom scales, six single-item symptom scales, and a financial impact scale.^39^ Scores range from 0 to 100 points, with a high score on the global QoL and functioning scales indicating a high QoL/high level of functioning and a high score on the symptom scales indicating a high level of symptomatology.^39^

In this study, the global QoL scale was chosen as the main outcome of HRQoL, as previously recommended.^39^ Additionally, following the recommendations of the EORTC Quality-of-Life Group, the QLQ-C30 summary score (SumSc) was used to supplement the 15-outcome profile generated by the QLQ-C30.^40^

### Secondary Outcomes

#### Exercise Capacity

Exercise capacity was assessed using the incremental shuttle walk test (ISWT) following the protocol described by Singh et al.^41^

#### Handgrip Strength

Handgrip strength was assessed using the Jamar Plus+ Dynamometer (Performance Health, Nottinghamshire, UK) following the standardized method recommended by the American Society of Hand Therapists.^42^

#### Five-Times Sit-to-Stand Test (5STS)

For the 5STS, patients were instructed to rise from a standardized armless chair (0.41–0.45 m high) to a standing position five times as quickly as possible without using their hands for support. The test was completed after the fifth repetition, and the time needed to perform the test was recorded using a stopwatch to the nearest 0.01 s.^43^

#### Postoperative LOS

The LOS was defined as the number of days patients spent in the hospital after surgery. Data were collected from the electronic medical records.

#### Exercise Adherence

Exercise adherence was measured as the percentage of total planned training volume completed, based on the patient’s exercise diary.^30,44^

#### Safety

Safety was assessed by collecting exercise-related adverse events during weekly phone calls. These were defined as any unfavorable or unexpected events associated with exercise training during or within 24 h after a training session.^45,46^ The Common Terminology Criteria for Adverse Events version 5 was used to categorize the severity of adverse events.^47^ An adverse event resulting in hospitalization, persistent or significant disability, or death was classified as a serious adverse event.^46,48^

### Sample Size Estimation

As suggested by evidence-based guidelines for sample size determination using the EORTC-QLQ-C30,^49^ the sample size was computed to detect a medium difference between groups in the global QoL scale corresponding to an effect size (Cohen’s d) of 0.5, from baseline to 1 month after surgery. Sample size calculation was performed using G*Power (version 3.1.9.2; Franz Faul, University of Kiel, Kiel, Germany), assuming a repeated-measures within-between interaction design with a minimal statistical power of 80% (significance level, 0.05), a correlation of 0.3 between pre- and post-surgery measures,^50^ and an effect size (*f*) of 0.25 (*f* = d/2). A sample size of 38 patients (19 per group) was retrieved. To account for a dropout rate of approximately 20%,^30^ a total sample size of 46 patients was estimated.

### Statistical Analysis

All data were analyzed using the SPSS package for Windows (version 27; IBM Corporation, Chicago IL, USA) according to the intention-to-treat principle. Tests were two-sided, and a *p* value lower than 0.05 was considered statistically significant. The normality of the data was assessed using the Shapiro-Wilk test. Baseline characteristics were compared between groups using independent samples *t* tests or the Mann-Whitney *U* test for continuous variables and using the chi-square test or Fisher’s exact test for categorical variables.

The primary analysis used a factorial repeated-measures analysis of variance (ANOVA) to test the effects of group across time on global QoL, exercise capacity, and handgrip strength (normally distributed data). If a significant group × time interaction was found, within-group differences were assessed by one-way repeated-measures ANOVA, and between-group differences were evaluated using independent-samples *t* tests. For non-normally distributed data (hospital LOS, 5STS, QLQ-C30 summary score, functioning and symptoms scales), within-group differences were assessed using Friedman’s test, and between-group differences were analyzed using the Mann-Whitney *U* test.

Additionally, the proportion of patients with clinically relevant changes in each domain of HRQoL from baseline to post-surgery was presented. These changes were determined for each scale by the minimal important difference for lung cancer patients.^51^ The proportion of patients who reported a clinically meaningful deterioration in HRQoL from baseline to post-surgery was compared between groups using the chi-square test.

## Results

**Fig. 2:**
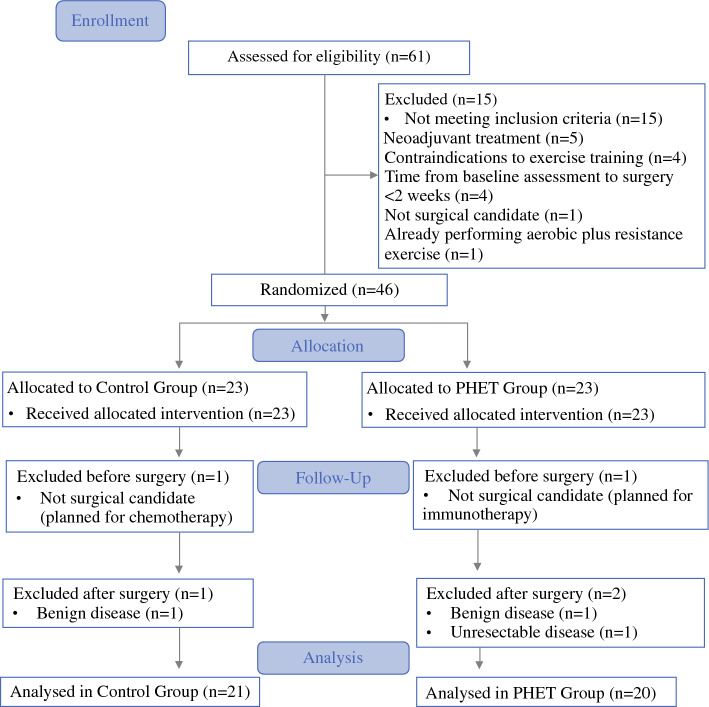
CONSORT flow diagram. CONSORT, Consolidated Standards of Reporting Trials; PHET, preoperative home-based exercise training

Between September 2022 and May 2023, 61 patients were screened for eligibility, and 46 were deemed eligible (Fig. 2). All 46 eligible patients (100%) were recruited for the study and randomly assigned to the PHET or CG. Five patients were excluded after randomization because they did not receive surgical treatment (*n* = 2), had no lung malignancy (*n* = 1), or had their tumor declared unresectable (*n* = 1). Hence, 41 patients were included in the intention-to-treat analysis (20 PHET patients and 21 CG patients).

Baseline demographic and clinical characteristics were balanced between the groups (Table 1). The patients had a mean age of 68.1 ± 9.3 years, were predominantly male (68.3%), had a diagnosis of adenocarcinoma (65.9%), and had tumor stage IA (58.5%). Most of the patients underwent lobectomy (79.1%) via video-assisted thoracoscopic surgery (80.5%). The mean time between the baseline assessment and surgery was 27.5 ± 8.5 days in the CG and 28.2 ± 7.9 days in the PHET group (*p* = 0.794).

**Table 1:** Participant demographic and clinical characteristics

Variable	All participants (*n* = 41) *n* (%)	CG (*n* = 21)* n* (%)	PHET (*n* = 20)* n* (%)	*p* Value
Mean age (years)	68.1 ± 9.3	68.7 ± 10.3	66.4 ± 7.2	0.668
Mean BMI (kg/m^2^)	26.5 ± 3.7	26.9 ± 3.6	26.5 ± 3.1	0.438
Sex (males)	28 (68.3)	15 (71.4)	13 (65)	0.658
*Educational level (years)*				0.505
<10	29 (70.7)	16 (76.2)	13 (65)	
≥10	12 (29.3)	5 (23.8)	7 (35)	
*Smoking status*				0.124
Current	16 (39)	10 (47.6)	6 (30)	
Former	14 (34.1)	4 (19)	10 (50)	
Never	11 (26.8)	7 (33.3)	4 (20)	
Smoking (mean packs/year)^a^	44.3 ± 24	39.4 ± 22.8	48.9 ± 24.8	0.279
*Tumor histologic subtype*				0.131
Adenocarcinoma	27 (65.9)	11 (52.4)	16 (80)	
Squamous cell carcinoma	6 (14.6)	5 (23.8)	1 (5)	
Carcinoid	7 (17)	4 (19)	3 (10)	
Pleomorphic carcinoma	1 (2.4)	1 (4.8)	0 (0)	
*Pathologic tumor stage*				0.567
IA	24 (58.5)	14 (66.7)	10 (50)	
IB	5 (12.2)	1 (4.8)	4 (20)	
IIA	1 (2.4)	0 (0)	1 (5)	
IIB	5 (12.2)	3 (14.3)	2 (10)	
IIIA	6 (14.6)	3 (14.3)	3 (15)	
*Comorbidities*				
Hypertension	24 (58.5)	14 (66.7)	10 (50)	0.222
COPD	10 (24.4)	4 (19)	6 (30)	0.484
Diabetes	9 (22)	7 (33.3)	2 (10)	0.130
History of myocardial infarction	4 (9.8)	1 (4.8)	3 (15)	0.343
Other	9 (22)	4 (19)	5 (25)	0.719
Median Charlson Comorbility Index^b^ (IQR)	4 (4–6)	5 (4–6)	5 (4–5.8)	0.968
*Mean pulmonary function*				
FVC (% predicted)	90.3 ± 17.7	86.6 ± 21.2	94.7 ± 11.4	0.169
FEV_1_ (% predicted)	84.9 ± 18	86.6 ± 20.2	83 ± 15.5	0.540
DLCO (% predicted)	72.6 ± 17.1	75.2 ± 18	69.6 ± 16.1	0.328
*Resection degree*				1.000
Lobectomy	34 (79.1)	17 (81)	17 (85)	
Bilobectomy	2 (4.7)	1 (4.8)	1 (5)	
Wedge resection	5 (11.6)	3 (14.3)	2 (10)	
*Surgical approach*				0.545
VATS	33 (80.5)	18 (85.7)	15 (75)	
Open surgery	7 (17.1)	3 (14.3)	4 (20)	
RATS	1 (2.4)	0 (0)	1 (5)	

*CG*, control group; *PHET*, preoperative home-based exercise training; *BMI*, body mass index; *COPD*, chronic obstructive pulmonary disease; *IQR*, interquartile range; *FVC*, forced vital capacity; *FEV*_1,_ forced expiratory volume in 1 s; *DLCO*, diffusion lung capacity for carbon monoxide; *VATS*, video-assisted thoracoscopic surgery; *RATS*, robot-assisted thoracoscopic surgery

^a^Pack-years only calculated for patients who previously smoked or currently smoke

^b^Scores range from 0 to 24, with higher scores indicating greater comorbidities

### Adherence and Safety

The mean duration of the PHET was 3.6 ± 0.2 weeks, and the patients completed a mean of 9.8 ± 3.1 sessions of aerobic exercise and a mean of 6.7 ± 2.3 sessions of resistance exercise (Table S4). The mean adherence rate was 103% ± 19.8% for aerobic training and 92.1% ± 33.1% for resistance training (Table S4). Six patients (30%) reported exercise-related adverse events (grade 1), predominantly leg muscle soreness (*n *= 4) (Table S3). No serious adverse events were observed.

### Primary Outcome: Effects on HRQoL

#### Global QoL Scale

**Fig. 3:**
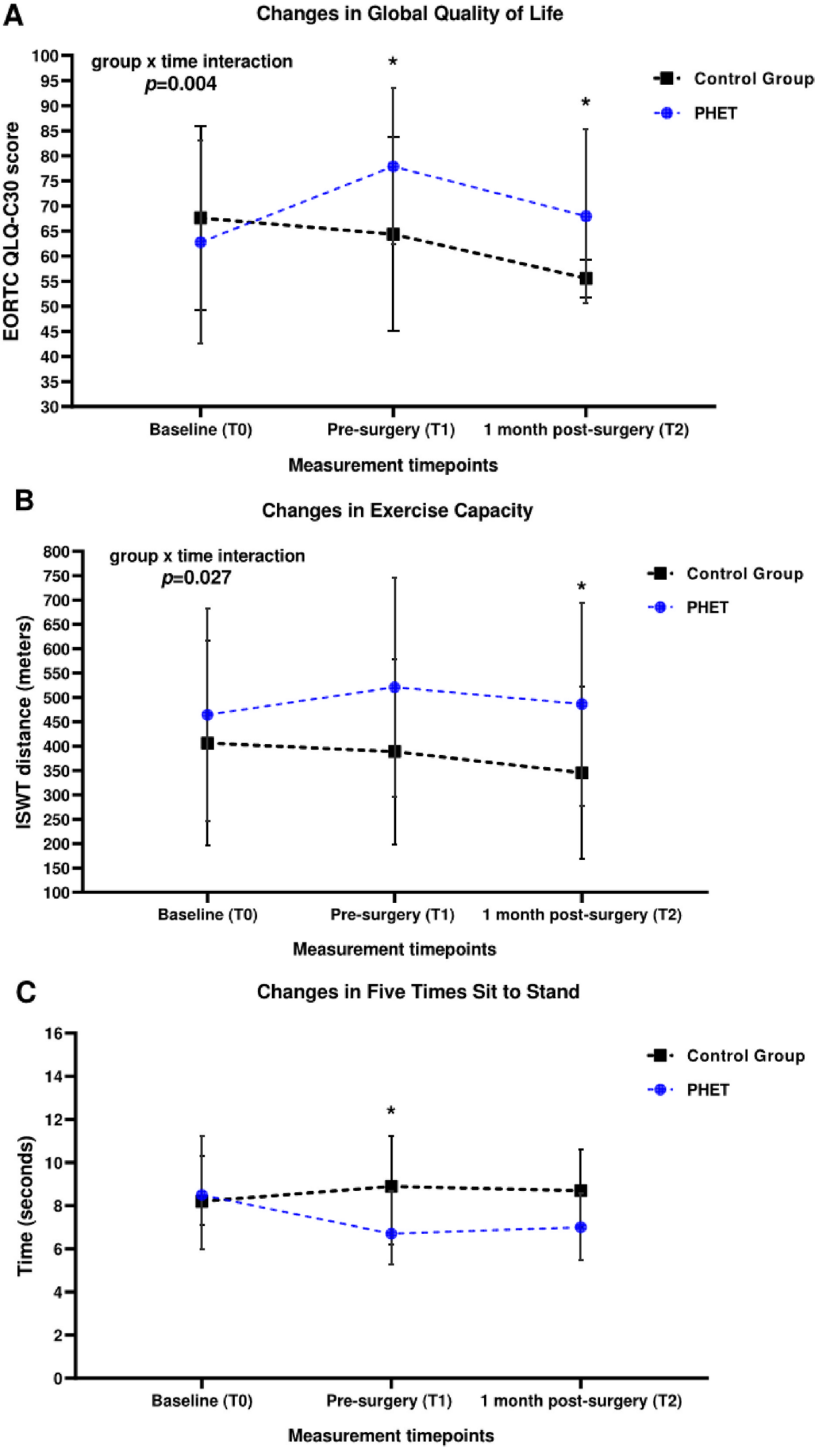
**A** Changes in Global Quality of Life scale across the study period. Data are expressed as means ± standard deviations. *Significant difference (*p* < 0.05) between groups (independent-sample *t* test). **B** Changes in exercise capacity across the study period. Data are expressed as means ± standard deviations. *Significant difference (*p* = 0.019) between groups (independent-sample *t* test. **C** Changes in five-times sit-to-stand across the study period. Data are expressed as median (interquartile range). *Significant difference (*p* = 0.041) between groups (Mann-Whitney *U* test). EORTC QLQ-C30, European Organization for the Research and Treatment of Cancer Quality of Life Questionnaire

**Fig. 4:**
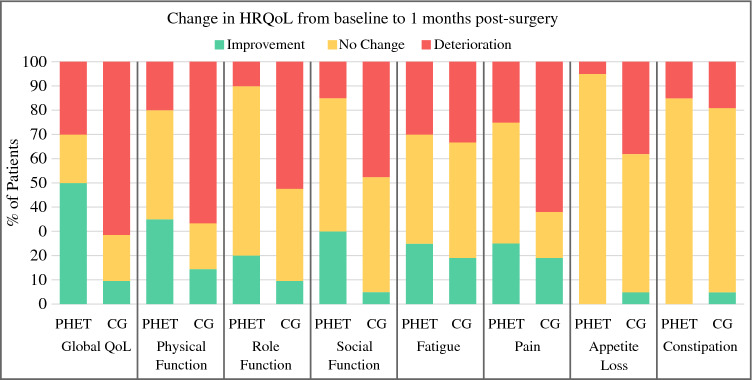
Changes in health-related quality of life from baseline to 1 month after surgery based on minimal important difference. HRQoL, health-related quality of life; QoL, quality of life; PHET, preoperative home-based exercise training; CG, control group. Minimal important difference for improvement: global health status (5 points), physical function (6 points), social function (6 points), role function (9 points), fatigue (6 points), pain (9 points), appetite loss (8 points), constipation (13 points). Minimal important difference for deterioration: global health status (−5 points), physical function (−7 points), social function (−5 points), role function (−9 points), fatigue (−9 points), pain (−12 points), appetite loss (8 points), constipation (−10 points).^51^

A significant group × time interaction was found in global QoL (*F*_2,38_ = 6.571*; p *= 0.004; Fig. 3A). Significant and clinically relevant differences between groups were found in global QoL before surgery (mean difference, 13.5 points; 95 % CI, 2.4–24.6 points; *p *= 0.019) and 1 month after surgery (mean difference, 12.4 points; 95 % CI, 1.3–23.4 points; *p *= 0.029), favoring PHET. Compared with baseline, 6 patients (30%) in the PHET group and 15 patients (71.4%) in the CG reported clinical deterioration in global QoL (*p *= 0.013; Fig. 4).

#### QLQ-C30 Functioning Scales

Significant differences between groups were found in physical function before surgery (*p *= 0.002) and after surgery (*p *= 0.020), favoring PHET. In addition, significant differences between groups were found in role function (*p *= 0.013) and emotional function (*p *= 0.045) after surgery, favoring PHET (Table 2).

**Table 2:** Changes in health-related quality of life and physical performance at the three time points of the study

Variable		Baseline (T0)	Pre-surgery (T1)		1-Month post-surgery (T2)		
EORTC-QLQ-C30 scale^a^	Group	Mean ± SD or median (IQR)	Mean ± SD or median (IQR)	Between-group differences *p v*alue^b^	Mean ± SD or median (IQR)	Between-group differences *p v*alue^b^	Within-group differences *p* value^b^
Global QoL^c^	PHET	62.8 ± 20.2	77.9 ± 15.6	**0.019**	68 ± 17.3	**0.029**	**0.018**^**d**^
	CG	67.6 ± 18.4	64.4 ± 19.3		55.6 ± 3.8		**0.006**^**e**^
Physical function^f^	PHET	93 (80–98.3)	96.5 (88.5–100)	**0.002**	87 (80–100)	**0.020**	0.075
	CG	87 (73.5–93)	87 (70–93)		73 (56.5–93)		**0.007**^**e**^
Role function^f^	PHET	100 (83–100)	100 (100–100)	0.925	100 (100–100)	**0.013**	0.630
	CG	100 (83–100)	100 (100–100)		83 (50–100)		**< 0.001**^**c**^
Social function^f^	PHET	100 (71–100)	100 (83–100)	0.853	100 (87.3–100)	0.130	0.607
	CG	100 (100–100)	100 (83–100)		100 (67–100)		**0.013**^**c**^
Emotional function^f^	PHET	75 (58–83)	83 (75–92)	0.523	92 (77–100)	**0.045**	**0.003**^**d,g**^
	CG	83 (71–96)	83 (67–92)		83 (62.5–92)		0.846
Cognitive function^f^	PHET	91.5 (83–100)	100 (83–100)	0.122	91.5 (83–100)	0.489	0.196
	CG	100 (83–100)	83 (67–100)		83 (67–100)		0.273
Fatigue^f^	PHET	11 (0–22)	0 (0–11)	**0.047**	11 (0–22)	0.200	0.072
	CG	11 (0–33)	11 (0–22)		22 (0–38.5)		0.624
Pain^f^	PHET	17 (0–17)	0 (0–17)	0.130	8.5 (0–17)	**0.041**	**0.049**
	CG	17 (0–17)	17 (0–25)		33 (8.5–50)		**0.024**^**c**^
Dyspnea^f^	PHET	0 (0–0)	0 (0–24.8)	0.930	0 (0–33)	0.662	0.444
	CG	0 (0–0)	0 (0–16.5)		0 (0–33)		0.651
Nausea and vomiting^f^	PHET	0 (0–0)	0 (0–0)	0.960	0 (0–0)	0.311	0.444
	CG	0 (0–0)	0 (0–0)		0 (0–0)		0.607
Insomnia^f^	PHET	33 (0–67)	(0–33)	0.450	0 (0–33)	0.927	0.973
	CG	0 (0–33)	0 (0–33)		0 (0–33)		0.088
Appetite loss^f^	PHET	0 (0–0)	0 (0–0)	0.679	0 (0–0)	**0.024**	1.000
	CG	0 (0–0)	0 (0–0)		0 (0–67)		**0.011**
Constipation^f^	PHET	0 (0–0)	(0–0)	0.598	(0–33)	0.481	0.050
	CG	0 (0–0)	0 (0–0)		0 (0–16.5)		0.223
Diarrhea^f^	PHET	0 (0–0)	0 (0–0)	0.544	0 (0–0)	0.563	1.000
	CG	0 (0–0)	0 (0–0)		0 (0–0)		1.000
Summary score^f^	PHET	90.5 (80.5–93)	93.5 (89.3–96)	0.071	92 (86–96.75)	**0.032**	**0.022**^**d**^
	CG	91 (86.5–95)	90 (88–93)		88.5 (74.5–92)		**0.024**^**e**^

Physical performance	Group	Mean ± SD or median (IQR)	Mean ± SD or median (IQR)	Between-group differences *p v*alue	Mean ± SD or median (IQR)	Between-group differences *p v*alue	Within-group differences *p* value
Incremental shuttle walk test (m)^c^	PHET	464. 5 ± 218.6	521.1 ± 225.1	0.051	486.3 ± 207.6	**0.027**	**0.016**^**d**^
	CG	406.4 ± 209.8	388.8 ± 190		345.5 ± 176.7		**0.045**^**e**^
Handgrip strength, right hand (kg)^c^	PHET	32.2 ± 9.3	31.8 ± 10	0.354	29.9 ± 7.8	0.630	0.116
	CG	28.4 ± 11.5	28.5 ± 11.7		28.4 ± 11.7		0.808
Handgrip strength, left hand (kg)^c^	PHET	30.6 ± 9.3	31 ± 10.4	0.294	28.4 ± 7.7	0.568	0.082
	CG	27 ± 10.2	27.5 ± 10.5)		26.7 ± 10.1		0.469
Five-times sit-to-stand (s)^f^	PHET	8.5 (6–11.2)	6.7 (5.3–8.7)	**0.041**	7 (5.5–8.6)	0.121	**<0.001**
	CG	8.2 (7.1–10.3)	8.9 (6.2–11.2)		8.7 (7–10.6)		0.892

EORTC-QLQ-30, European Organization for Research and Treatment of Cancer Quality of Life Questionnaire C30; SD, standard deviation; IQR, interquartile range; QoL, quality of life; PHET, preoperative home-based exercise training; CG, control group

^a^Higher scores for global QoL scale, summary score, and functioning scales (physical–cognitive function) denote better quality-of-life/functionality; higher scores for symptom scales (fatigue–diarrhea) denote worse symptomatology

^b^Bold numbers indicate significant differences (*p* < 0.05)

^c^Results are expressed as mean (standard deviation)

^d^Indicates a significant improvement from baseline to pre-surgery

^e^Indicates a significant decline from baseline to 1-month post-surgery

^f^Results are expressed as medians (interquartile ranges)

^g^Indicates a significant improvement from baseline to 1 month post-surgery

From baseline to 1 month after surgery, the proportion of patients who reported clinical deterioration was significantly lower in the PHET group compared with the CG in physical function (PHET [*n *= 4, 20%] vs CG [*n *= 14, 66.7%]; *p *= 0.004), role function (PHET [*n *= 2, 10%] vs CG [*n *= 11, 52.4%]; *p *= 0.006), and social function (PHET [*n *= 3, 15%] vs CG [*n *= 10, 47.6%]; *p *= 0.043) (Fig. 4).

#### QLQ-C30 Symptom Scales

Before surgery, significant differences between groups were found in fatigue (*p *= 0.047), favoring PHET. At 1 month after surgery, significant differences between groups were found in pain (*p *= 0.041) and appetite loss (*p *= 0.024), favoring PHET (Table 2). From baseline to 1 month after surgery, the proportion of patients who reported clinical deterioration in these symptoms was significantly lower in the PHET group than in the CG (pain: PHET [*n *= 5, 25%] vs CG [*n *= 13, 61.9%], *p *= 0.028; appetite loss: PHET [*n *= 1, 5%] vs CG [*n *= 8, 38.1%], *p *= 0.020) (Fig. 4).

#### QLQ-C30 Summary Score

Before surgery, no differences between groups were found in SumSc (*p *= 0.071). At 1-month after surgery, the PHET group reported a significantly better SumSc than the CG (*p *= 0.032; Table 2).

### Secondary Outcomes

#### Effects on Physical Performance

*Exercise Capacity, Handgrip Strength, and 5STS.* A significant group × time interaction was found in exercise capacity (*F*_2,36_ = 6.448; *p *= 0.004). Before surgery, there was a trend for significant differences between groups in exercise capacity (*p* = 0.051), favoring PHET. At 1 month after surgery, the PHET group had significantly better exercise capacity than the CG (mean difference, 147.4 m; 95% CI, 17.3–264.2 m; *p* = 0.027; Fig. 3B). In addition, the two groups differed significantly in 5STS before surgery (median difference, −1.8 s; 95% CI, −0.1 to −3.7 s; *p *= 0.041), favoring PHET (Fig. 3C). No significant between-group differences in handgrip strength were found (*p *> 0.05; Table 2).

#### Effects on Postoperative LOS

No significant between-group differences were found in postoperative LOS (PHET: 5 [interquartile range {IQR}, 3.3–11] vs CG: 4 [IQR, 3–5.5]; *p* = 0.187).

## Discussion

The novel findings of this study were that PHET is clinically effective in improving the HRQoL of lung cancer patients awaiting surgical resection and preventing its deterioration after surgery. Specifically, the PHET group showed improvements in preoperative global QoL, physical function, and fatigue compared with the CG. More importantly, these beneficial effects were maintained postoperatively, with the patients who participated in PHET reporting a significantly and clinically better global QoL than the patients in the CG. Furthermore, 1 month after surgery, the PHET group exhibited significantly better physical, emotional, and role functions, together with fewer symptoms of pain and appetite loss than the CG.

These findings are clinically relevant given that the preoperative HRQoL of lung cancer patients is significantly worse than in the general population,^52^ and surgical treatment causes even further impairments in short- and long-term HRQoL.^2,4,8^ To the best of our knowledge, this is the first multicenter RCT demonstrating that a short-term preoperative home-based exercise intervention (3–4 weeks) can improve HRQoL after lung cancer surgery.^11^

The beneficial effects observed on global QoL, physical function, and fatigue are aligned with the strong evidence that exercise training is effective in improving these domains of HRQoL among cancer patients.^10,53,54^ However, much of this evidence is provided by clinical trials conducted with breast cancer survivors^10^ and with patients undergoing (neo-)adjuvant treatment or after oncologic treatment,^33^ limiting the generalization of the findings.^10^

In the context of lung resection, only a few trials have examined the effects of prehabilitation on postoperative HRQoL. Ferreira et al.^55^ found that patients who performed prehabilitation consisting of a multimodal intervention (home-based aerobic and resistance exercise plus nutritional counseling and anxiety-reduction strategies) had significantly better global QoL, physical function, and mental function 4 weeks after surgery than patients who started the intervention postoperatively.

These results are partially aligned with a clinical trial involving patients who underwent resection for benign and malignant lung disease.^19^ The findings showed that preoperative exercise training improved physical function 3 months after surgery compared with usual care, although no significant differences in mental function were observed.^19^ Collectively, these findings corroborate our results by indicating that the benefits of preoperative exercise training in terms of global QoL and physical function persist after lung cancer surgery. However, further research is warranted to examine the effects of this intervention on mental and emotional function after surgery.

Another important finding of the current study was that the patients in the PHET group had significantly better role function and fewer symptoms of pain and appetite loss after surgery than those in the CG. These results are impactful given that most lung cancer patients had a clinical deterioration in role function and experienced significantly more pain and appetite loss 1 month after surgery compared with preoperative levels.^2–4^ Previous studies on this topic suggest that exercise training is effective in improving the role function of cancer patients.^33,56^ However, conflicting evidence exists regarding the effect of this intervention on pain and appetite loss.^57,58^ Because no RCTs have been conducted with patients awaiting lung cancer surgery, it is not possible to compare the current results with those of other studies. This highlights the need for future studies to investigate the effect of preoperative exercise training on these HRQoL domains.

The secondary purpose of this study was to determine the effects of PHET on physical performance and LOS. We found that PHET resulted in significantly better performance in the preoperative 5STS test than CG. This represents an important finding because poor performance in the STS test before lung cancer surgery has been linked to a greater risk of postoperative complications.^59^ Additionally, in line with a previous trial,^60^ we found that PHET prevented the decline in postoperative exercise capacity, which is clinically important because patients’ exercise capacity declines significantly after surgery.^61^ Because exercise capacity is independently associated with HRQoL after curative treatment for lung cancer,^62^ the beneficial effects of PHET on this outcome may have contributed to patients experiencing a better HRQoL, possibly by improving their ability to perform activities of daily living.

Although previous meta-analyses showed that preoperative exercise training reduces the LOS after lung cancer surgery,^15,17^ no significant differences between groups were observed in the current study. However, it should be noted that most studies included in these meta-analyses were single-center trials conducted in a facility-based setting,^15,17^ emphasizing the need for future multicenter RCTs to verify the effects of home-based exercise training on this outcome.

The high adherence rate and absence of serious adverse events during the intervention are consistent with the results of previous feasibility trials.^30,63^ Taken together, these results indicate that PHET is well tolerated by patients and may overcome barriers that hinder access to prehabilitation, namely, transportation problems.^23^

The strengths of this study included the multicenter design, the comprehensive synthesis of the effects from PHET in the different domains of HRQoL, and the high recruitment, retention, and adherence rates. The study limitations included the small sample, although it was adequately powered for the primary outcome; exercise adherence assessed on the basis of self-reported diaries, which are susceptible to social desirability bias;^64^ and the exclusion of patients who received neoadjuvant treatment, limiting the generalization of findings to these subgroups of individuals who have lower preoperative aerobic capacity than those not receiving this treatment.^65^

## Conclusion

The current study showed that PHET improves HRQoL before lung cancer surgery and prevents its deterioration after surgery. In this study, PHET was particularly beneficial for global QoL and physical function, which clinically improved preoperatively and remained significantly better after surgery compared with CG. In addition, the results demonstrated that PHET can effectively improve postoperative exercise capacity.

The findings of this study support the integration of PHET into the perioperative care of lung cancer patients to prevent the detrimental impact of surgery on HRQoL and exercise capacity. Future studies with larger samples are needed to clarify the effects of PHET on pain, appetite loss, and emotional and role functions after surgery.

### Supplementary Information

Below is the link to the electronic supplementary material.

Supplementary file1 (PDF 433 KB)
