# Supplementary material for: Effect of Preoperative Home-Based Exercise Training on Quality of Life After Lung Cancer Surgery: A Multicenter Randomized Controlled Trial
Source: Ann Surg Oncol. 2023 Nov 7;31(2):847–59. doi: 10.1245/s10434-023-14503-2 (PMC10761542; doi:10.1245/s10434-023-14503-2)
Supplement: Supplementary file 1 — Supplementary file1 (PDF 433 KB) [file 10434_2023_14503_MOESM1_ESM.pdf]

Table S1. Contraindications to the preoperative exercise program

**Major signals of symptoms suggestive of cardiovascular, metabolic and renal disease**	Pain or; discomfort in the chest, neck, jaw, arms, or other areas that may result from ischemia;<br>Dizziness or syncope;<br>Orthopnea or paroxysmal nocturnal dyspnea;<br>Ankle edema;<br>Palpitations,<br>Intermittent claudication;<br>Known heart murmur;<br>Unusual fatigue or shortness of breath with usual activities
**Severe arterial hypertension**	Resting systolic blood pressure > 180 mmHg or diastolic blood pressure > .100 mmHg.
**Cardiovascular disease**	Uncontrolled symptomatic heart failure;<br>Severe ischemia of the cardiac muscle upon exertion;<br>Recent pulmonary embolism (< 3 months ago) causing severe hemodynamic strain;<br>Thrombophlebitis;<br>Acute pericarditis or myocarditis;<br>Active endocarditis;<br>Hypertrophic cardiomyopathy<br>Hemodynamically serious aortic stenosis or mitral valve stenosis;<br>Myocardial infarction less than 3 months before the start of the training program;<br>Uncontrolled cardiac dysrhythmias causing symptoms or hemodynamic compromise;<br>Suspected or known dissecting aneurysm:<br>Cerebrovascular disease resulting in transient ischaemic attacks<br>Other acute cardiovascular event less than 3 months before the start of the training program (*e.g.*, acute heart failure, acute coronary syndrome, cardiogenic shock or hypertensive crisis)
**Respiratory disease**	Pneumonia;<br>Uncontrolled asthma;
**Metabolic disease**	Uncontrolled metabolic disease (e.g., diabetes, thyrotoxicosis, or myxedema)
**Orthopedic disorders**	Lower-limb amputation without prosthesis,<br>Orthopedic disorders exacerbated by exercise training
**Other comorbidities**	Alzheimer disease;<br>Serious psychiatric disorders;<br>Severe visual or hearing disorders,<br>Uncorrected medical conditions, such as anemia or important electrolyte imbalance<br>Other mental or physical impairment leading to inability to exercise adequately and safety
**Other signals or symptoms**	Fever;<br>Dyspnea while speaking.<br>Desaturation with exercise training

Table S2 Consensus on Exercise Reporting Template

Section/Topic	Item	Checklist item	Description
**WHAT: Materials**	1	Detailed description of the type of exercise equipment (e.g. weights, exercise equipment such as machines, treadmill, bicycle ergometer etc)	The exercises were performed using bodyweight (calisthenics), a step platform and free-weights of one and two kilograms.
**WHO: Provider**	2	Detailed description of the qualifications, teaching/supervising expertise, and/or training undertaken by the exercise instructor.	Physical therapist with qualifications (master degree in sport science and personal trainer certification) and expertise in exercise prescription in cancer patients.
**HOW: Delivery**	3	Describe whether exercises are performed individually or in a group	Exercises were performed individually by each patient.
	4	Describe whether exercises are supervised or unsupervised and how they are delivered	Sessions of walking and resistance training were performed at home (unsupervised). The exercise program included a weekly telephone supervision.
	5	Detailed description of how adherence to exercise is measured and reported.	Exercise adherence was measured as the percentage of total completed to planned training volume, based on patients' exercise diaries Training volume was determined for each session of aerobic exercise (walking time (minutes)) and resistance exercise (sum of number of sets × number of repetitions for each exercise). Then, the training volume of all sessions was summed to quantify the total completed training volume
	6	Detailed description of motivation strategies.	The exercise program was designed to match patients' preferences regarding exercise modality (walking) and setting (home-based program).<br>Positive and prescriptive feedback was given during weekly telephone supervision to reinforce patients behavioral change and encourage the continuation of the training program. The exercise program had a flexible schedule, allowing patients who could not perform an exercise session in the planned day, to compensate in another day of the week.
	7	Detailed description of the decision rule(s) for determining exercise progression.	The following criteria for progression or regression in the exercise training dose were considered:

		Detailed description of how the exercise program was progressed.	Criteria to regression: If patients reported a perceived exertion higher than 5 (strong) during walking or resistance training, they were advised to split the 30 minutes of walking in bouts of 10 minutes per day and/or to decrease the volume load in resistance training (reduce the number of repetitions from 15 to 10 and/or sets from two to one).<br>Criteria to progression: If patients reported a perceived exertion lower than 3 (moderate) during walking or resistance training, they were advised to increase the duration of aerobic exercise in five minutes per session and/or increase the volume load in resistance training (increase the number of repetitions from 15 to 20 and/or the number of sets from two to three).<br>If exercise dose was well tolerated during week 1 and 2 (i.e., no serious or persistent adverse events and rate of perceived exertion between 3-5 on Borg RPE CR-10 scale) patients were advised to increase the dose of walking and resistance training:<br>Walking: Increase the duration in 10 minutes (after 2nd week).<br>Resistance training: Increase the number of sets (3 sets of 15 repetitions after the 2nd week)
	8	Detailed description of each exercise to enable replication (e.g. photographs, illustrations ,<br>video etc)	The exercised prescribed were: sit-to-stand, step-up, calf raises, glute bridge, shoulder press and biceps curls. Illustrations and QR codes to access videos of each exercise are provided in Figure S1.
	9	Detailed description of any home program component (e.g. other exercises, stretching etc)	The exercise program was entirely performed at home.
	10	Describe whether there are any non-exercise components (e.g. education, cognitive behavioural therapy, massage etc)	The intervention contained an educational session.
	11	Describe the type and number of adverse events that occurred during exercise	A total of 6 patients (30%) reported exercise-related adverse events (Grade 1), predominantly leg muscle soreness (n=4).
**WHERE: location**	12	Describe the setting in which the exercises are performed	The exercise program was performed in a home-based environment.
**WHEN, HOW MUCH: dosage**	13	Detailed description of the exercise intervention including, but not limited to, number of exercise repetitions/sets/sessions, session duration, intervention/program duration etc.	Exercise dosage, based on F.I.T.T principle (Frequency, intensity, time and type), is detailed in Methods (PHET group). Exercise sessions also included a warm-up (joint mobility exercises) and a cool down period (thoracic stretching and an exercise of breathing

			control). To resemble daily clinical practice in the institutions involved, the length of the exercise program was adjusted based on the waiting times for surgery.
	14	14a Describe whether the exercises are generic (one size fits all) or tailored whether tailored to the individual<br>14b Detailed description of how exercises are tailored to the individual.	The exercise prescription followed the guidelines from the most recent international consensus about exercise prescription in oncology. These guidelines suggest that combined moderate intensity aerobic (20-30 minutes per week; 2-3 sessions per week) and resistance training (2 sets of 8-15 reps; 2-3 sessions per week) is effective to improve health-related quality of life in cancer patients.[8]<br>The exercise dose prescribed was tailored based on patients' musculoskeletal limitations. (Table S3).
	15	Describe the decision rule for determining the starting level at which people commence an exercise program (such as beginner, intermediate, advanced etc)	Resistance exercise: The initial dose prescribed was 2 sets of 15 repetitions, rate of perceived rate of perceived exertion between 3–5 (moderate to strong) on the Borg CR-10 scale, twice weekly. The starting external load (free-weights) was determined based on the following criteria: 1) no exercise-related pain when performing the exercise 2) allowing the completion of 15 repetitions with a correct technique; 3) rate of perceived exertion between 3–5 on the Borg CR-10 scale. When a criterion was not achieved, the decision was:<br>1st: External load adjustments (decrease or increase 1kg);<br>2st: Volume load adjustments (decrease or increase 5 repetitions and, if required, decrease or increase the number of sets).<br>Aerobic exercise: The initial dose prescribed was 30 minutes of walking, rate of perceived rate of perceived exertion between 3–5 on the Borg RPE CR-10 scale, thrice weekly. Adaptations in walking duration (minutes) were made in patients with musculoskeletal pain exacerbated by walking or to achieve a rate of perceived exertion between 3–5 on the Borg RPE CR-10 scale.
		16a Describe how adherence or fidelity to the exercise intervention is assessed/measured.<br>16b Describe the extent to which the intervention was delivered as planned.	Adherence to the exercise intervention was measured based compliance with the prescribed training volume, using patients' records in exercise diaries. Results are described in detail for each patient, integrating information about individual adaptations.

Table S3 Exercise adherence and adverse events (individual patient data)

**Patient no.**	**Length** (weeks)	**Type**	**Attendance rate**	**Total training volume**	**Adherence rate**†	**Weekly training volume** (average)	**Average intensity** (RPE* and external load)	**Missed sessions**	**Adverse events**
**1**	4 weeks	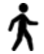	**100%** (12 of 12 sessions)	**460 min.**	**110%**	**115 min.**	3.8		**Grade 1:** Arthralgia (shoulder join) during shoulder press; mild pain, not limiting ADL
		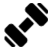	**100%** (8 of 8 sessions)	**1800 reps**	**100%**	**450 reps**	3.3		
**2**	3 weeks	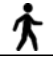	**100%** (8 of 8 sessions)	**270 min**	**100%**	**90 min**	3		**Grade 1:** Leg muscle soreness after walking; mild symptoms, not limiting ADL
		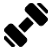	**100%** (6 of 6 sessions)	**1260 reps**	**100%**	**420 reps**	3<br>1-2 kg		
**3***	3 weeks	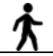	**78%** (7 of 9 sessions)	**210 min.**	**70%**	**70 min.**	3	N=3 sessions<br>Reasons: Preparation and recovery of a mediastinoscopy	**No adverse events**
		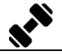	**83%** (5 of 6 sessions)	**288 reps**	**29%**	**96 reps**	4<br>1kg		
**4****	4.5 weeks	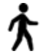	**100%** (14 of 14 sessions**)**	**563 min.**	**113%**	**125 min.**	**5**		**Grade 1:** Arthralgia (shoulder join) during shoulder press; mild pain, not limiting ADL<br>**Grade 1:** Leg muscle soreness after walking; mild symptoms, not limiting ADL
		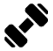	**100%** (9 of 9 sessions)	**3600 reps.**	**174%**	**800 reps**	2<br>2kg		

Table S3 Exercise adherence and adverse events (individual patient data) (continued)

**Patient no.**	**Length** (weeks)	**Type**	**Attendance rate**	**Total training volume**	**Adherence rate**†	**Weekly training volume** (average)	**Average intensity** (RPE* and external load)	**Missed sessions**	**Adverse events**
**5**	4 weeks	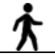	**75%**<br>(9 of 12 sessions)	**300 min.**	**71%**	**75 min.**	5.5	N=5 sessions<br>Reasons: Joint stiffness (patient had rheumatoid arthritis)	**No adverse events**
		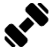	**75%**<br>(6 of 8 sessions)	**1260 reps**	**70%**	**315 reps**	5<br>2 kg		
**6**	3 weeks	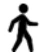	**100%**<br>(8 of 8 sessions)	**360 min.**	**133%**	**120 min.**	3.4		**No adverse events**
		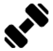	**133%**<br>(8 of 6 sessions)	**1620 reps**	**129%**	**540 reps**	3.2<br>1 kg		
**7**	2 weeks	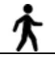	**100%**<br>(6 of 6 sessions)	**210 min.**	**117%**	**105 min.**	3		**No adverse events**
		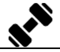	**100%**<br>(4 of 4 sessions)	**720 reps**	**100%**	**360 reps**	3<br>2 kg		
**8**	4.5weeks	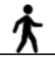	**92%**<br>(12 of 13 sessions)	**580 min.**	**114%**	**129 min.**	4	N=2 sessions<br>Reasons: Preparation and recovery of a mediastinoscopy	**No adverse events**
		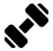	**89%**<br>(8 of 9 sessions)	**1710 reps**	**79%**	**380 reps**	4<br>1-2kg		

TableS3 Exercise adherence and adverse events (individual patient data) (continued)

**Patient no.**	**Length** (weeks)	**Type**	**Attendance rate**	**Total training volume**	**Adherence rate**†	**Weekly training volume** (average)	**Average intensity** (RPE* and external load)	**Missed session**	**Adverse events**
**9**	4 weeks	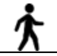	**92%**<br>(11 of 12 sessions)	**390 min**	**93%**	**98 min.**	3.6	N=1<br>Reason: No time	**No adverse events**
		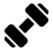	**100%**<br>(8 of 8 sessions)	**1710 reps**	**95%**	**428 reps**	4<br>2 kg		
**10*** **	5 weeks	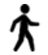	**100%**<br>(15 of 15 sessions)	**570 min.**	**106%**	**114 min.**	1.6		**No adverse events**
		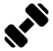	**100%**<br>(10 of 10 sessions)	**2340 reps**	**100%**	**468 reps**	2.4<br>1 kg		
**11**	2.5 weeks	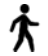	**63%**<br>(5 of 8 sessions)	**150 min.**	**68%**	**60 min.**	2	N=6<br>Reasons: Medical appointments. lack of time and bad weather	**No adverse events**
		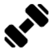	**40%**<br>(2 of 5 sessions)	**360 reps.**	**36%**	**144 reps.**	2<br>1-2 kg		
**12**	3 weeks	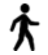	**100%**<br>(8 of 8 sessions)	**310 min.**	**115%**	**103 min.**	5		**Grade 1**: Leg muscle soreness after resistance exercise; mild symptoms, not limiting ADL
		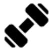	**100%**<br>(6 of 6 sessions)	**1260 reps**	**100%**	**420 reps**	4<br>1-2 kg		
**13**	3 weeks	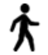	**100%**<br>(9 of 9 sessions)	**330 min.**	**122%**	**110 min.**	3		**No adverse events**
		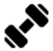	**100%**<br>(6 of 6 sessions)	**1440 reps**	**100%**	**480 reps**	4<br>2 kg		

Table S3 Exercise adherence and adverse events (individual patient data) (continued)

**Patient no.**	**Length** (weeks)	**Type**	**Attendance rate**	**Total training volume**	**Adherence rate**†	**Weekly training volume** (average)	**Average intensity** (RPE* and external load)	**Missed sessions**	**Adverse events**
**14**	5.5 weeks		**88%** (15 of 17 sessions)	**505 min.**	**81%**	**92 min.**	3.5	N=2 sessions<br>Reason: Bad weather	**No adverse events**
			**100%** (11 of 11 sessions)	**1825 reps**	**70%**	**332 reps**	4.5<br>1 kg		
**15**	2 weeks		**100%** (6 of 6 sessions)	**180 min.**	**100%**	**90 min.**	3		**Grade 1**: Leg muscle soreness after walking; mild symptoms, not limiting ADL
			**100%** (4 of 4 sessions)	**900 reps.**	**125%**	**450 reps.**	3.7<br>2 kg		
**16*****	5 weeks		**87%** (13 of 15 sessions)	**705 min.**	**131%**	**141 min.**	3	N=3 sessions<br>Reasons: Medical appointments and bad weather.	**No adverse events**
			**90%** (9 of 10 sessions)	**1980 reps.**	**85%**	**396 reps**	3<br>1-2 kg		
**17**§	4 weeks		**92%** (11 of 12 sessions)	**370 min.**	**88%**	**93 min.**	3	N=2 sessions<br>Reason: Covid-19.	**Grade 1** Back pain (lower back) during glute bridge; mild symptoms, not limiting ADL<br>**Grade 1:** Back pain (upper back) after resistance exercise; mild symptoms, not limiting ADL
			**88%** (7 of 8 sessions**)**	**1200 reps**	**67%**	**300 reps**	3<br>1kg		
**18**	2 weeks		**133%** (8 of 6 sessions)	**225 min.**	**125%**	**113 min.**	3		**No adverse events**
			**100%** (4 of 4 sessions)	**720 reps**	**100%**	**360 reps**	5<br>2 kg		

Table S3 Exercise adherence and adverse events (individual patient data) (continued)

**Patient no.**	**Length** (weeks)	**Type**	**Attendance rate**	**Total training volume**	**Adherence rate**[†]	**Weekly training volume** (average)	**Average intensity** (RPE* and external load)	**Missed sessions**	**Adverse events**
**19**	4 weeks		**92%** (11 of 12 sessions)	**390 min.**	**93%**	**98 min.**	3.3	N=2 sessions<br>Reason: Medical examinations	**No adverse events**
			**88%** (7 of 8 sessions)	**1980 reps**	**110%**	**495 reps**	2.3<br>2 kg		
**20**	3 weeks		**88%** (7 of 8 sessions)	**290 min.**	**112%**	**97 min.**	3.4	N=2 sessions<br>Reason: Medical examinations	**No adverse events**
			**83%** (5 of 6 sessions)	**810 reps**	**64%**	**270 reps**	3.4<br>2 kg		

Legend: ADL (activities of daily living); obs. (observation); min. (minutes); kg (kilograms); reps (repetitions); RPE (rate of perceived exertion)

Aerobic exercise; Resistance exercise

Higher than the planned training volume Planned training volume Lower than the planned training volume

* Required dose reduction before the initiation of the training program due to very strong fatigue symptomatology during the resistance exercises (Borg CR-10= 7/10). The volume load prescribed was 1 set of 8 reps (external load=1kg).

** Performed interval aerobic training (3 minutes of walking interspersed with 1 minute of running) to achieved the prescribed intensity (RPE 3-5 on Borg CR-10).

***Glute bridge was changed by standing hip flexion.

[§] Patient not performed glute bridge due to low back pain (preexisting symptoms that increased during this exercise)

.[†] Ratio of total completed to planned training volume, expressed as a percentage

Table S4 Exercise adherence

Variable	Aerobic exercise	Resistance exercise
**Number of sessions,** mean (SD)	9.8 (3.1)	6.7 (2.3)
**Adherence rate**[a] **(%),** mean (SD)	103 (19.8)	92.1 (33.1)
**Weekly training volume** (min/reps), mean (SD)	101.8 (20.1)	400.4 (147.5)
**Intensity (**RPE)[b], median (IQR)	3.5 (3.1-3.8)	3.3 (3-4)

Legend: IQR (interquartile range); kg (kilograms); min. (minutes); no. (number); reps (repetitions); RPE (rate of perceived exertion); SD (standard deviation); aerobic exercise; resistance exercise.

[a]Ratio of total completed to planned training volume, expressed as a percentage. Training volume was determined for each session of aerobic exercise (walking time (minutes)) and resistance exercise (sum of number of sets × number of repetitions for each exercise). Then, the training volume of all sessions was summed to quantify the total completed training volume; [b]Based on Borg Category Ratio scale (0–10 scale)

Exercise 1: Step up

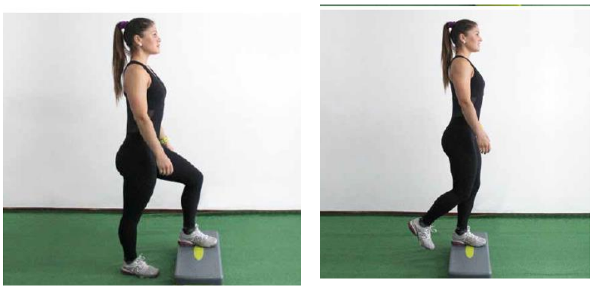

Exercise 2: Glute bridge

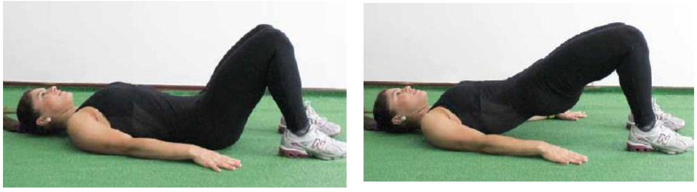

Exercise 3: Sit to stand

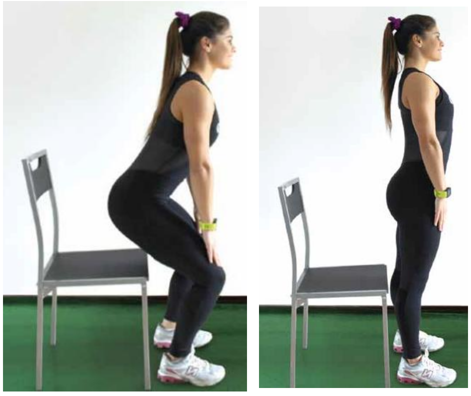

Exercise 4: Shoulder Press

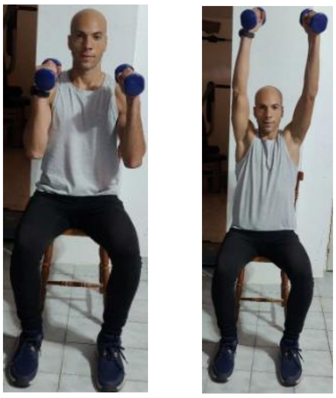

Exercise 5: Biceps Curl

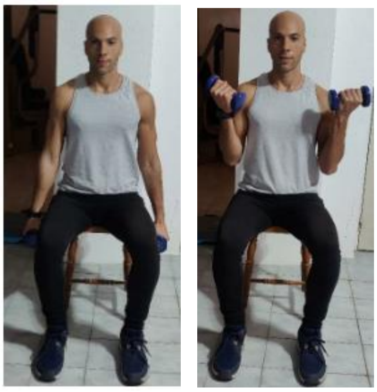

Exercise 6: Calf Raises

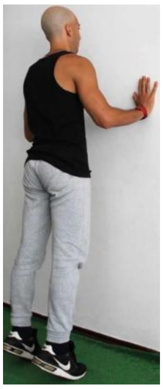

Figure S1. Home-based resistance exercises
